# Online support groups for head and neck cancer and health-related quality of life

**DOI:** 10.1007/s11136-017-1575-8

**Published:** 2017-04-17

**Authors:** Eamar Algtewi, Janine Owens, Sarah R. Baker

**Affiliations:** The Academic Unit of Dental Public Health, School of Clinical Dentistry, University of Sheffield, 19 Laremont Crescent, Sheffield, S10 2TA UK

**Keywords:** Head and neck cancer, Online support, Quality of life, Adjustment, Empowerment

## Abstract

**Purpose:**

To investigate the association between using online support groups (OSGs) and health-related quality of life (HRQoL), and the psychosocial factors that may influence this association among individuals with head and neck (H&N) cancer.

**Method:**

A sample of 199 persons with H&N cancer using four OSGs completed an online questionnaire using six pre-validated measures for social network, self-efficacy, anxiety and depression, adjustment, empowerment and quality of life. In addition, socio-demographic as well as illness-related and OSGs-related information was collected.

**Results:**

Participants who had better HRQoL had been using OSGs for a longer time than those who had worse HRQoL (*B* = 0.07, *p* < 0.05). Depression and adjustment were the only direct mediators in this association, whereas self-efficacy, anxiety and empowerment appeared as indirect mediators.

**Conclusion:**

Participation in OSGs was found to be associated to better HRQoL either directly or indirectly through decreasing depression, anxiety and the negative adjustment behaviours and increasing self-efficacy and empowerment of the users. The study presented a potential model of pathways linking OSG use and HRQoL for those with H&N cancer. However, the model needs to be tested in future longitudinal studies and the associations proposed need to be explored in greater detail.

## Introduction

Head and neck (H&N) cancer includes malignant tumours arising from the mucosa of the upper aerodigestive tract from nasopharynx to larynx including the oral cavity, i.e. pharynx, lip and oral cavity and larynx [1]. This group of cancers is amongst the six most prevalent cancers in the world [2] and some of them are associated with high mortality rates [3].

Previous literature has suggested that H&N cancer can have a negative influence on quality of life (QoL) through negative physical and psychosocial impacts including swallowing difficulties, impaired speech, problems in physical appearance, anxiety, depression, fear of relapse and loss of self-esteem e.g. [4]. However, it has also been suggested that some factors may mitigate or aggravate the impact of cancer generally on a patient’s psychological wellbeing [4, 5]. For example, there are many strategies that people diagnosed with H&N cancer can employ which may influence health outcomes. One such strategy is coping. In general, higher levels of adaptive coping have been found to be related to better QoL of AIDS patients [6]. Other psychological factors such as anxiety and depression have also been widely linked to reduced levels of QoL for cancer patients in general e.g. [7] and other non-cancerous conditions e.g. [8].

Self-efficacy has been shown to be related to better coping and relatively low levels of psychological distress in some chronic diseases like HIV [9]. In line with this, it has been found that people who receive social support have stronger self-efficacy beliefs, which subsequently may affect their health-related outcomes [10]. This mediating role of social support on psychological outcomes has been found in relation to a number of health conditions e.g. [11]. Related to this, studies on social networks suggest that such networks can also be related to health-related quality of life (HRQoL) and, in addition, may have a mediating effect on anxiety and depression amongst cancer patients. For example, Michael et al. [12] found that social interaction at pre-diagnosis level was a significant factor in future HRQoL among women who experienced breast cancer. The evidence for social networks establishes that social isolation increases the risk of mortality after being diagnosed with breast cancer, with the buffering effect for reduction being provided by relatives or friends and participation in activities outside the home e.g. [13]. Social networks have also been found to play an important role in enhancing the coping ability of patients with laryngeal and hypopharyngeal cancer [14].

In the last few decades, an increasing number of people have explored the internet for support, information and advice related to many aspects of health [15], and there is an expanding number of online support groups (OSGs) available for different health conditions including cancer [16]. Online support groups may have many advantages over conventional face-to-face groups and may have several benefits to their users; for example, they have been found to be associated with reduction in levels of both physiological and psychological stress in their members [17]. These groups can increase social support by increasing self-esteem, personal empowerment and functional status and decreasing depression, feelings of helplessness, distress and social isolation e.g. [18–20].

It may be that people with H&N cancer, especially those who have impairment in speech or alteration in facial appearance, might find OSGs a suitable environment in which they can socialise and get support in more comfortable ways without feelings of embarrassment regarding their situation. Nevertheless, despite the high incidence of H&N cancer and its influence on the HRQoL of patients and the beneficial role of OSGs, there has been no research, to date, on the association between OSGs and HRQoL for people with H&N cancer or on the psychosocial factors that might mediate this association.

Therefore, the aim of this study was to examine the association between using online support groups and health-related quality of life and examine the psychosocial factors (social network, self-efficacy, anxiety, depression, adjustment and empowerment) that may influence this association.

## Method

### Procedure

A thorough Internet search was established to identify OSGs for people with H&N cancer. The most common Internet search tools (Google, Yahoo, Bing and MSN) and Facebook were searched using the terms ‘online support group’ and ‘head and neck cancer’ and the relevant synonyms, connotations and denotations of these. OSGs were then selected based on two main criteria [6]:

1. The OSG was active with at least 25 message threads posted to the group within the past 30 days.
2. The group contained at least 50 members.

**Fig. 1:**
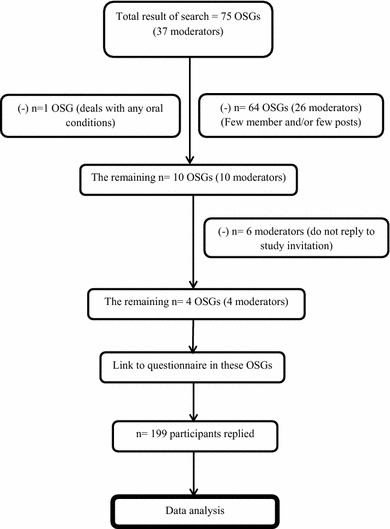
Online support groups’ sample profile

OSGs that focused on oral conditions other than H&N cancer or were published in languages other than English were excluded. The initial search generated 75 OSGs; following application of the exclusion criteria, only 10 OSGs remained (see Fig. 1).

Following the necessary ethical approval, moderators from these groups were contacted explaining the research study and asking for permission to recruit participants from their group. Out of the 10 moderators contacted, four replied and provided permission. A message was then placed on the discussion boards of each of these four groups explaining the aim and objectives of the study. The questionnaire was provided in the message in the form of hyperlink which contained the information sheet, consent form and instruction leaflet on how to complete the questionnaires. The aims and objectives of the study, inclusion criteria and confidentiality of data and rights of participant as well as instructions about the survey were explained in the information sheet which appeared first when the participants clicked on the study link, and informed consent was sought from participants before they were able to complete the questionnaire. There was an email address for participants to ask further questions should they so wish. After that, participants were directed automatically to the questionnaire where they could submit their responses online. All questions within the questionnaire were set so that they were mandatory to answer. The participant could not proceed to the next page or submit the questionnaire without all the questions having been answered. Therefore, only 100% complete responses were received. Participants were those who had been diagnosed with H&N cancer at any point during their life and used H&N cancer-related OSGs. There were no financial or material motivations provided to the participants and the measured variables were based on current status of the participants.

The sample size for the study was calculated using the software ‘G-power’ [21]. The sample size calculation was based on a medium effect size of *f* ^2^ = 0.15, statistical power of 95%, a significance level of 0.05 and 15 potential variables in the analysis. From this, the sample size calculated was 199 participants.

Ethical approval was sought from the University Research Ethics Committee (UREC). The project followed the ethical guidelines of the British Psychological Society [22], including informed consent and confidentiality.

## Measures

### Demographics

Data on participant’s age, gender, country, marital status and income status were collected by questions adapted from the UK Census [23].

### Illness-related variables

Participants were asked to report cancer site, stage, treatment type, treatment stage and time since diagnosis. Cancer site was categorised into three main groups: lip and mouth cancer, throat cancer and vocal cords, whereas cancer stage was recorded as Stage I, Stage II, Stage III and Stage IV according to Tumour Node Metastasis (TNM) staging system [24], and in both situations there was an extra choice for people who “were not sure”. In addition, questions about treatment type were modified from a questionnaire used for cancer by Susan and Clingman [25], in which participants could tick as many as they liked (e.g. chemotherapy, radiation and surgery).

### Online support group use

Respondents were asked to estimate the duration of using OSGs (in months), frequency of use per month (in days), frequency of use per day (in hours), membership of OSG (yes/no), duration of membership (in months) and approximate number of posted and replied messages. To investigate the utility of these measures to represent the use of OSGs, a bivariate analysis (Pearson’s correlations for the continuous measures and Spearman’s correlations for the discrete measures) was conducted between HRQoL and each measure. Only “Duration of using OSG” (*p* = 0.02) was significantly related to HRQoL, whereas the remaining variables were not significant. Therefore, this measure was chosen to assess the association between the use of OSG and HRQoL.

### Psychosocial variables

*Social network* was measured using The Berkman–Syme Social Network Index (BSNI), an 11-item self-reported questionnaire [26]. The BSNI is designed to assess the type, size, closeness and frequency of contacts in a respondent’s current social network. The BSNI considers both relative importance and number of social ties among the four groups and unites this material into a summary measure ranging from 0 to 4 [27]. BSNI allows researchers to classify people into four stages of social connection: socially isolated (people with few close contacts, fewer than six friends or relatives, not married and no membership in either community or groups church), moderately isolated, moderately integrated and socially integrated. The most isolated category belongs to scores summed: 0 or 1. The BSNI is a valid and reliable index in assessing patient’s social network as a factor known to influence morbidity and mortality in people with chronic disease [28, 29].

*Self*-*efficacy* was measured using The Cancer Behaviour Inventory-brief version (CBI-B) 12-item validated questionnaire [30] used widely as a measure of self-efficacy for coping with cancer, derived from the longer 33-item version [31]. Participants responded to each question on a 9-point Likert scale, with a possible score of “1” = ‘Not at all confident’ to “9” = ‘Totally confident’ reflecting the degree of confidence the patient has that he or she can perform that particular coping behaviour. Previous studies have indicated that the CBI-B has good internal reliability (*α* = 0.84) and construct validity [30].

*Anxiety and Depression* were measured using Hospital Anxiety and Depression Scale (HADS) 14-item questionnaire [32], which is commonly used to determine the levels of anxiety and depression that an individual is experiencing in both hospital and community settings. Seven of the items relate to anxiety and seven relate to depression. Each question has 4 possible responses ranging from 0 to 3. The maximum score is 21 (0–7 = Normal; 8–10 = Borderline abnormal; 11–21 = Abnormal). The scale has demonstrated good reliability [33].

*Adjustment to cancer* was measured using The Mini-Mental Adjustment to Cancer (MINI-MAC) 29-item questionnaire [34]. The Mini-MAC items are rated on a 4-point Likert scale ranging from “Definitely does not apply to me” (1) to “Definitely apply to me” (4) and measures patient’s experiences at present. It also has five subscales: Helpless–Hopeless, Anxious Preoccupation, Cognitive Avoidance, Fighting Spirit and Fatalism. Higher scores indicate higher endorsement in these coping strategies. Several studies evaluating the psychometric properties of the Mini-MAC scale have supported its validity and reliability of all five subscales of this questionnaire e.g. [35].

*Empowerment* was measured using the Empowering Processes Scale (EPS) [36]. This scale has 39 items measuring 4 dimensions of empowering processes (receiving social support, finding positive meaning, receiving useful information, helping others). Participants were asked the frequency in which each event took place in the OSG on a 5-point Likert scale ranging from 1 = never to 5 = very often, with higher scores indicating higher levels of empowering processes. The reliability of the original empowering processes scale was satisfactory (Cronbach’s alpha ranged from 0.70 to 0.95). The Cronbach’s alpha of the four subscales ranged from 0.87 (helping others) to 0.95 (finding positive meaning) [36].

*Health*-*related quality of life* was measured using the 12-item University of Washington Quality of Life Questionnaire (UW-QoL) [37, 38]. Each question has from 3 to 5 answers, with participants choosing one appropriate answer that applies to them. Each of the domain-specific items is scored from 0 (worst QoL) to 100 (best QoL). The ‘composite’ score is created by averaging the scores from the 12 items, and therefore the possible total scores ranged from 0 to 100. The validity of UW-QOL questionnaire version 4 has been assessed in many studies using translated version to other languages e.g. [39], and the overall internal consistency ranged between Cronbach’s alpha values of 0.73 and 0.84.

### Statistical analysis

In order to address the aims of the study, a series of regression analyses were carried out. The first research question to be addressed was as follows:

#### Is there an association between OSG use and health-related quality of life?

A linear regression analysis (Enter method) was conducted using the duration of using OSGs measure as the predictor variable (*duration of use*) and the HRQoL score as the outcome variable. The data met the assumption of linearity, homoscedasticity, normality of the residuals and reliability of measurement needed for regression analysis.

The second research question to be addressed was as follows:

#### Is the association between OSG use and HRQoL mediated by social network, self-confidence, anxiety, depression, adjustment or empowerment?

**Fig. 2:**
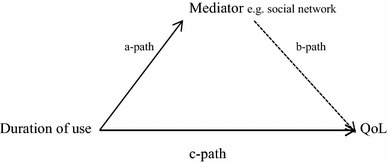
Example of process of mediation between OSG (duration of use) and QoL

To investigate this, the mediation regression tests (Enter method) outlined by Baron and Kenny [40] were followed. Baron and Kenny proposed a model (Fig. 2) which includes three paths: the direct path (c-path) between the predictor (e.g. duration of use) and the dependent variable (e.g. HRQoL) and the indirect paths (a-path and b-path) that include the mediator (e.g. Social network).

Baron and Kenny suggest that if the mediation requirements hold, using the example above, duration of use should be a significant predictor of HRQoL (c-path) and duration of use should be a significant predictor of the mediator variable (a-path). In addition, there should be a significant association between the mediator variable and HRQoL (b-path) while controlling for duration of use. Baron and Kenny’s model also indicates that when the mediator (e.g. social network) and duration of use are used simultaneously to predict HRQoL, the previous association between duration of use and HRQoL should be reduced or become non-significant. Following the Baron and Kenny’s model, the mediating role of the psychosocial variables was tested using SPSS using a tool by Hayes [41].

## Results

### Participants

 A total of 199 respondents living with H&N cancer completed the online questionnaire for the study. As can be seen from Table 1, the mean age of the participants was 54.34 with 70% of them over 50 years. There were 101 (50.8%) females and 98 (49.2%) males originating from 6 countries with the majority from the USA (56.2%). The majority of participants were married or living with their partners and most were satisfied with their income status.

**Table 1:** Demographics, Illness-related variables and OSGs variables

Variable	Number	%
Age		
10–19	1	0.50
20–29	1	0.50
30–39	13	5.53
40–49	43	21.60
50–59	81	40.70
60–69	48	24.12
70–79	12	6.05
Sex		
Male	98	49.24
Female	101	50.75
Country		
USA	112	56.28
The UK	45	22.61
Australia	22	11.05
Canada	15	7.53
New Zealand	4	2.01
France	1	0.50
Marital Status		
Married/living with a partner	145	72.86
Widowed	9	4.52
Single	30	15.07
Prefer not to say	2	1.00
Other	16	8.04
Income status		
Totally insufficient	25	12.56
Somewhat insufficient	40	20.10
Sufficient for essential needs	91	45.72
More than sufficient	43	21.60
Cancer site		
Lip and mouth	76	38.19
Throat	81	40.70
Vocal cords	34	17.08
I am not sure	8	4.02
Cancer stage		
Stage I	22	11.05
Stage II	14	7. 03
Stage III	27	13.56
Stage IV	94	47.23
I am not sure	42	21.10
Treatment type		
Chemotherapy	102	51.25
Radiation	177	88.94
Surgery	154	77.38
Acupuncture/oriental medicine	18	9.04
Naturopathy	5	2.51
Herbology or nutritional consulting	10	5.02
Online support groups	131	65.82
Colour, art or music therapy	5	2.51
Spiritual healing	9	4.52
Prayer	80	40.20
Meditation or self-healing	25	12.56
Psychological counselling	32	16.08
Face-to-face support groups	32	16.08
Massage or other bodywork	33	16.56
I am not sure	2	1.00
Other	16	8.04
Treatment stage		
Pre-diagnosis	1	0.50
Pre-treatment	3	1.50
Under-treatment	19	9.54
Post-treatment	174	87.43
I am not sure	2	1.00
Time since diagnosis		
≤1 year	45	22.61
>1–<5 years	91	45.72
≥5–<10 years	44	22.11
≥10 years	13	6.53
Unknown	6	3.01
Duration of using OSGs		
≤1 year	81	40.70
>1–<5 years	92	46.23
≥5–<10 years	19	9.54
≥10 years	4	2.01
Missed data	3	1.50
Days per month		
0–9	57	28.64
10–19	34	17.08
20–29	32	16.08
Daily	74	37.18
Missed data	2	1.00
Hours per day		
0–<1	46	23.11
1–<2	88	44.22
2–<3	22	11.05
3–<4	6	3.01
4–<5	2	1.00
≥5	20	10.05
Missed data	15	7.53
Number of posted messages		
1–9	72	36.18
10–49	74	37.18
50–99	11	5.52
100–499	11	5.52
500–1000	4	2.01
>1000	0	0.00
Missed data	27	13.56
Number of replied messages		
1–9	53	26.63
10–49	55	27.63
50–99	23	11.55
100–499	37	18.59
500–1000	7	3.51
>1000	4	2.01
Missed data	20	10.05

### Illness-related and OSGs variables

As can be seen from Table 1, the most frequently reported types of cancer were throat and lip and mouth cancer, and most of the participants (60.7%) were in the advanced stages of cancer (Stages III and IV). The majority of participants were treated with radiation, surgery and chemotherapy, and the vast majority of them (87.4%) were in the post-treatment stage. Approximately 30% of the participants were diagnosed more than 5 years ago (mean: 53.7 months).

It can also be observed from Table 1 that more than half of the participants had been using OSGs for more than one year (mean: 31.4 months). The data also indicated that around half of the participants were frequent users of OSGs (more than 20 days per month) with 37.2% of participants being daily users (mean: 18.1). The majority of participants (70%), when they used the OSG, did so for at least 1 hour per day and some of them (10%) for extended periods of time (5 h +).

The reliability, mean and standard deviation for all of the psychosocial measures in the study are shown in Table 2.

**Table 2:** Reliability, mean and standard deviation of the questionnaires

Variable	Measure	Cronbach’s alpha	Mean	SD	Range (min–max score)	Number of items
Social network	BSNI	0.76	1.45	0.88	4 (0–4)	11
Self-efficacy	CBI –B	0.86	81.80	16.13	76 (32–108)	12
Anxiety and depression	HADS	0.91	13.44	7.89	21 (0–39)	14
	Anxiety	0.88	7.88	4.41	21 (0–21)	7
	Depression	0.86	5.56	4.17	18 (0–18)	7
Adjustment	MINI-MAC	0.82	69.34	10.44	61 (39–100)	29
Empowerment	EPS	0.96	131.52	26.63	148 (39–187)	39
Quality of life	UW-QOL	0.79	68.17	14.74	75.83 (19.58–95.42)	12

With regard to the first Research Question, i.e. is there an association between OSG use and HRQoL, the results indicated that the duration of use was significantly related to the HRQoL of participants (*B* = 0.07, *t* = 2.32, *p* = 0.02), such that those participants who had higher HRQoL scores had been using OSGs for a longer period of time (*df* = 195, *F* = 5.41), this model explained 2.7% in HRQoL outcome (*R* ^2^ = 0.027).

The second research question examined the mediating role of social network, self-confidence, anxiety, depression, adjustment and empowerment in the association between OSG use and HRQoL. Using the methodology of Baron and Kenny outlined in the statistical analysis section, in the first step, testing the c-path, OSG usage was positively associated with HRQoL (see Table 3). The a-paths for each possible mediator were then tested (i.e. their association with OSG usage) and the results are shown in Table 3.

**Table 3:** Relationship between OSG duration of use, QoL and the six proposed mediators

	*B*	*t*	*p*
c-path			
QoL	0.07	2.32	0.02
a-path			
Social network	0.00	0.94	0.34
Self-efficacy	0.08	2.35	0.02
Anxiety	−0.03	−3.72	0. 00
Depression	−0.02	−3.10	0.00
Adjustment	−0.07	−3.18	0.00
Empowerment	0.13	2.41	0.01
b-path			
Self-efficacy	−0.11	−1.63	0.10
Anxiety	0.15	0.57	0.57
Depression	−2.46	−8.44	0.00
Adjustment	−0.33	−3.38	0.00
Empowerment	−0.03	−1.16	0.24

From Table 3, it can be seen that there was a significant association between duration of use and self-efficacy, anxiety, depression, adjustment and empowerment. Social network was not related to OSG use and so, according to the requirements of Baron and Kenny, could not be considered a mediator of duration of use on HRQoL.

In the final step of the regression model, testing the b-path, whilst controlling for duration of use, the results indicated that only depression and adjustment were associated with HRQoL (see Table 3). Therefore, self-efficacy, anxiety and empowerment, according to the requirements of the Baron and Kenny’s model, cannot be considered as direct mediators of OSG on HRQoL.

The results also indicated that the direct effect of duration of use on HRQoL became non-significant (*p* = 0.96) when controlling for depression and adjustment, thus suggesting full mediation. Therefore, the association between longer duration of use and better HRQoL was mediated by people having lower levels of negative adjustment behaviour (e.g. fatalism) toward their H&N cancer and lower levels of depression (*df* = 198, *F* = 42.67, *p* = 0.02), and this model explained 52.5% in HRQoL outcome (*R* ^2^ = 0.525).

Given that depression and adjustment were the only possible direct mediators in the association between the use of OSGs and HRQoL, exploratory analyses were carried out to investigate whether the remaining factors that had significant a-path with the duration of use (anxiety, self-efficacy and empowerment) had any indirect mediating role. This was achieved by identifying whether they mediated the association between OSGs and depression and/or adjustment, and subsequently by identifying the mediators in the association between OSGs and each of anxiety, self-efficacy and empowerment.

**Fig. 3:**
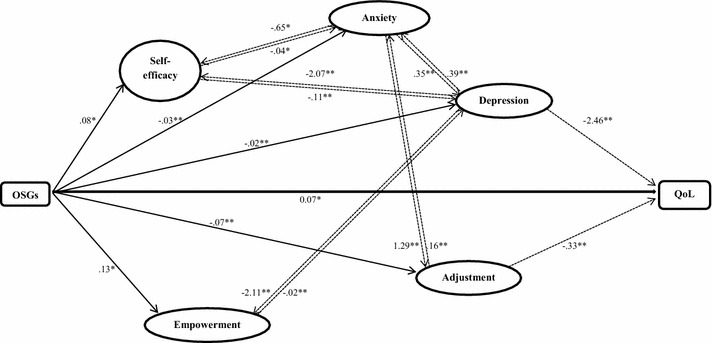
Summary of the mediating process in the relationship between OSGs and QoL, depression, adjustment and anxiety. **Correlation is significant at the 0.01 level (2-tailed); *Correlation is significant at the 0.05 level (2-tailed). *Solid arrows* c-paths or a-paths, *dotted arrows* significant b-paths, and *Numbers* regression coefficients (*B*)

This strategy had led to further six regression models (see “Appendix 1” Section). A summary model of the associations between the use of OSGs and HRQoL and the proposed mediators that resulted from these regression analyses (see Table 3 and “Appendix 1” Section) is shown in Fig. 3. In summary, self-efficacy, anxiety and depression were found to mediate each other in their associations with OSGs. Depression was also found to mediate and be mediated by empowerment. Similarly, anxiety was also found to mediate and be mediated by adjustment.

## Discussion

The main finding of this study was an association between using H&N cancer-related OSG and HRQoL of participants, such that longer use was linked to better HRQoL. Moreover, this association was mediated by depression and adjustment. Anxiety, self-efficacy and empowerment were found to have a role in the mediation process by mediating the association between the use of OSGs and depression and adjustment.

The findings of this study are consistent with previous literature that reports a link between OSG use and better HRQoL for HIV/AIDS [6, 42]. It is also consistent with a meta-analysis of 28 studies exploring the health-related outcomes associated with computer-mediated support groups [43].

Previous studies suggested that using OSGs including group communication and educational components lead to an improvement in HRQoL directly or indirectly through increasing the use of adaptive coping and decreasing the use of maladaptive coping [6, 42, 43]. In line with this, the findings of the current study suggested that depression, adjustment, self-efficacy, anxiety and empowerment, all act to mediate the association between duration of use and HRQoL directly or indirectly. The results relating to self-efficacy, adjustment and empowerment are in line with previous literature [6], in which the authors have investigated the possible mechanism through which participation in OSGs might encourage user empowerment for people living with HIV/AIDS. Findings of that study proposed that participation in OSGs results in empowering processes, which in turn have a positive influence on psychosocial outcomes as measured by coping, self-care self-efficacy and HRQoL. This consistency in findings might indicate that the mechanisms underlying the mediation process between using OSGs and HRQoL is similar despite differences in the nature of health condition under investigation.

With regard to the role of anxiety and depression, no previous studies have investigated the mediating role of anxiety and depression in the OSG-HRQoL association. However, some studies on different health conditions have found negative associations between using OSGs and depressive symptoms e.g. [47] as well as feelings of dental anxiety [48, 49]. Additionally, the literature indicated that depression and anxiety are widely known to have a negative association with at least one aspect of HRQoL in people with H&N cancer [50] or other cancers or non-cancerous health conditions e.g. [7].

The duration of using OSGs was also found to be directly related to depression, anxiety, adjustment, self-efficacy and empowerment. Those participants who had been using OSGs for a longer time had lower levels of anxiety and depression, lower negative adjustment (in terms of negative coping strategies such as helpless–hopeless, anxious preoccupation, avoidance and fatalism), higher levels of empowerment processes (in terms of receiving social support, finding positive meaning, receiving useful information and helping others) and a greater belief that they are capable of performing well (i.e. self-efficacy).

In the present study, there was no association between the duration of use and social network. It is possible that people with H&N cancer might be part of non-virtual social networks and receive social support from other sources, apart from OSGs.

Although all the proposed mediators were found to play a role in the association between OSGs and HRQoL (except social network), the results indicated that, while controlling for all the proposed mediators, only levels of depression and adjustment were found to be direct mediators, such that participants who had been using OSGs a longer time reported low levels of depression and adjustment and in turn reported better HRQoL. Nevertheless, whilst the other variables were not mediators of the OSG-HRQoL association, they were found to play a role in other associations within the pathway (see Fig. 3). In general, participants who had been using OSGs a longer time were less depressed, less anxious, had fewer tendencies for the negative adjustment behaviours, and confident that they were capable of performing, felt more empowered to cope with their illness and in turn had better HRQoL. Interestingly, although the worldwide incidence of oral cancer is more prevalent in males than females in the majority of countries [44], in our study the male:female ratio was almost equal. It may be, as has been reported previously, that women are more likely to use the Internet for the purposes of interpersonal communication [45] and are more interested generally in health-related topics [46].

There were a number of limitations in the current study which need to be noted when interpreting the findings. Most importantly, despite testing for mediation processes, the present study was cross-sectional. Whilst mediation was tested using techniques outlined within the literature [40], temporality cannot be assumed with cross-sectional data. The pathways proposed here are therefore exploratory and need to be tested longitudinally in future work. In the present study, variables such as depression and anxiety were measured at the time of taking the survey. That is, they were the participant’s current reported status. It is likely, however, that depression, for example, may change as a result of length of survivorship. A future longitudinal study should collect information on depression and the other time-varying variables before, during and immediately after treatment. In addition, from our data it is not possible to ascertain whether the use of OSGs improved HRQoL or vice versa; that is, people who had better HRQoL were more likely to use OSGs. Further, given that we found that more than half of participants had been using OSGs for 5 years or more, it may be that longer survivorship improved their HRQoL. A further longitudinal study would be needed to address this important question.

Participation in the study was optional, and therefore it may be that only people with positive experiences of OSGs agreed to participate, or perhaps those who were adjusting better to their condition or treatment. It may be that differing results would have been obtained if people who had different experiences or were poorly adjusted to their condition had been included. In addition, given the study’s online methodology, it was not possible to collect information on stage and exact site of the cancer, or types of treatment, from patient’s notes. Therefore, such information was reported by the participants themselves and remains unverifiable. It may be that differing results might have been obtained if this information were collected from an independent source (e.g. patient’s notes; clinician).

The study used an online survey because it felt that it might be a convenient way for collecting data from people who are in various geographic areas in the world and have access to the internet. However, the literature suggests that there are some disadvantages for online surveys, including issues of non-representativeness of the sample, low response rates, non-responses and lack of validity of the data [51]. In addition, the study recruited participants from the internet (OSGs) and this methodology can have drawbacks such as errors in self-reported demographics and the risk of self-selection bias, the possibility of duplicate or fraudulent responses and the inability, by the research team, to verify the cancer status of participants [52, 53]. However, given the absence of financial incentives and the length of the questionnaire, it seems unlikely that participants would duplicate their responses or misrepresent themselves as being a cancer patient [53]. As has been noted that there are a number of limitations when conducting online research, however, these authors conclude that whilst being aware of such limitations, online research can be a cost-effective method of recruiting and a very useful tool for exploring health-related issues.

The inclusion criteria for this study included people who used OSGs and had been diagnosed with H&N cancer at any point during their life. Indeed, the results of the study showed that the majority of participants (87.4%) were in their post-treatment stages. This strategy may have drawbacks in that people at different stages of cancer and/or its treatment may have different perspectives from each other and may differ from people who have already finished their treatment in terms of their use and association with OSGs, as well as their responses to the study questionnaires.

Although several measures were used to assess the use of OSGs, only the main outcome variable, duration of use, was found to have a significant association with HRQoL. Since this measure is reported by participants, it could be subject to self-report bias. It is also a rather crude measure and does not consider the level of participation within the group, including the number of messages posted and participant activity in different periods, or their daily use. Future studies should investigate more closely the association with the actual levels of participation in terms of posting messages as well as the daily and monthly rate of use.

Nevertheless, despite these limitations, the present study suggests an exploratory model of potential pathways linking OSG use and HRQoL for those with H&N cancer which could be investigated in future studies, with H&N cancer as well as other cancers or chronic health conditions.

There are a number of implications for health care professionals from these findings when considering their support for people with H&N cancer. Those professionals may want to encourage patients to use OSGs to seek support and information related to their condition. They may also provide help to make patients aware of the internet for support and about how to facilitate patients’ skills with this technology, or provide training themselves.

The findings of this research as well as previous literature [18, 44] suggest that most people who live with H&N cancer and most of the users of H&N cancer OSGs were older people. If we consider this, then we could argue that efforts should be directed toward providing access to the internet among those people, perhaps by providing them with access to equipment, training and free internet, as well as informing them about relevant OSGs and websites.

## Appendix 1: Results of subsequent regression mediation analysis

### Model 2: Mediators between OSGs and Depression

The proposed mediators were self-efficacy, anxiety, empowerment and adjustment.

**Table:**

	*B*	*t*	*p*
b-path			
Self-efficacy	−0.11	−7.65	0.00
Anxiety	0.35	5.62	0.00
Adjustment	0.01	0.42	0.67
Empowerment	−0.02	−2.99	0.00

Model fit: *df* = 198, *F* = 80.28, *p* = 0.00, *R* ^2^ = 0.623

### Model 3: Mediators between OSGs and adjustment

The proposed mediators were self-efficacy, anxiety, empowerment and depression.

**Table:**

	*B*	*t*	*p*
b-path			
Self-efficacy	−0.09	−1.86	0.06
Anxiety	1.29	7.27	0.00
Depression	0.09	0.42	0.67
Empowerment	−0.03	−1.49	0.13

Model fit: *df* = 198, *F* = 47.12, *p* = 0.00, *R* ^2^ = 0.493

### Model 4: Mediators between OSGs and anxiety

The proposed mediators were self-efficacy, depression, adjustment and empowerment.

**Table:**

	*B*	*t*	*p*
b-path			
Self-efficacy	−0.04	−2.27	0.02
Anxiety	0.40	5.62	0.00
Adjustment	0.17	7.27	0.00
Empowerment	0.01	1.70	0.09

Model fit: *df* = 198, *F* = 81.48, *p* = 0.00, *R* ^2^ = 0.627

### Model 5: Mediators between OSGs and self-efficacy

The proposed mediators were anxiety, depression, adjustment and empowerment.

**Table:**

	*B*	*t*	*p*
b-path			
Anxiety	−0.65	−2.27	0.02
Depression	−2.07	−7.65	0.00
Adjustment	−0.19	−1.86	0.06
Empowerment	−0.02	−0.67	0.50

Model fit: *df* = 198, *F* = 60.00, *p* = 0.02, *R* ^2^ = 0.553

### Model 6: Mediators between OSGs and empowerment

The proposed mediators were self-efficacy, anxiety, depression and adjustment.

**Table:**

	*B*	*t*	*p*
b-path			
Self-efficacy	−0.11	−0.67	0.50
Anxiety	1.15	1.70	0.09
Depression	−2.11	−2.99	0.00
Adjustment	−0.36	−1.49	0.13

Model fit: *df* = 198, *F* = 3.95, *p* = 0.01, *R* ^2^ = 0.075
